# A Multifaceted Evaluation Approach for Greek Native Neglected and Underutilized Forest Fruit Trees and Shrubs as Natural Sources of Antioxidants: Consolidating the Framework for Their Sustainable Agronomic Exploitation

**DOI:** 10.3390/plants12081642

**Published:** 2023-04-13

**Authors:** Eleftherios Karapatzak, Olga Dichala, Katerina Papanastasi, Ioannis Manthos, Ioannis Ganopoulos, Antonis Karydas, Anastasia V. Badeka, Ioanna S. Kosma, Dimitris Kyrkas, Paraskevi Yfanti, Nikolaos Nikisianis, Giorgos Patakioutas, Eleni Maloupa, Nikos Krigas

**Affiliations:** 1 Institute of Plant Breeding and Genetic Resources, Hellenic Agricultural Organization Demeter, 57001 Thessaloniki, Greece; 2 Laboratory of Food Chemistry, Department of Chemistry, University of Ioannina, 45110 Ioannina, Greece; 3 Department of Agriculture, School of Agriculture, University of Ioannina (UOI), 47100 Kostakii Arta, Greece; 4 Systems of Forest and Environmental Development (SYSTADA), 8 Amasia Str., 55133 Thessaloniki, Greece

**Keywords:** native plants, small fruits, antioxidant capacity, free radical scavenging capacity, natural compounds, neglected and underutilized species, forest germplasm, ex situ cultivation

## Abstract

Fruits from wild forest trees and shrubs represent a natural source of antioxidants against oxidative stress and a growing market for novel minor crops. This study presents a multifaceted approach which sets the basis for sustainable agronomic exploitation of selected Greek native germplasm of four traditional but neglected and underutilized forest fruit trees and shrubs, namely *Amelanchier ovalis* Medik., *Cornus mas* L., *Rosa canina* L., and *Sambucus nigra* L. The studied plant species are traditionally used in Greek ethnobotany but are currently neglected and underutilized in commercial terms, thus characterized as neglected and underutilized plant species (NUPs). The investigation includes new information on the evaluation of the ex situ cultivation of the Greek germplasm (three of the four focal NUPs), thus supplementing respective full datasets for their comparative evaluation based on four evaluation axes (documentation and molecular authentication of genotypes, phytochemical evaluation, asexual propagation via rooting of cuttings, and ex situ cultivation) after multi-year and multifaceted groundwork data previously acquired. Consecutively, the work includes feasibility and readiness timescale evaluation for the sustainable exploitation of each focal species based on existing literature and extant research experience. The feasibility for sustainable exploitation and readiness timescale evaluation results were very encouraging, showing high exploitation feasibility with an already achieved readiness timescale for *R. canina* and *S. nigra,* whereas *C. mas* and *A. ovalis* showed an achievable readiness in the short term. The comparative evaluation of the Greek native focal NUPs outlined the excellent potential of *R. canina*, *S. nigra*, and *A. ovalis*, and the high potential of *C. mas*. The results herein illustrate the very high fruit antioxidant potential (free radical scavenging activity) of all focal species, the diverse but effective asexual propagation capacity via cuttings at the species level, and summarize the results of a pilot cultivation trial set up in 2020 (still ongoing) outlining tree growth rates and the onset of fruit production among genotypes and species. Overall, the meta-analysis of previously published data in conjunction with new data generated herein may serve the sustainable exploitation of the studied NUPs.

## 1. Introduction

Oxidative stress and its detrimental effects on human health stemming from the imbalance between free radicals (reactive oxygen species) and antioxidants (molecules with free radical scavenging capacity) has to date received significant attention globally. To this end, intensive research efforts are currently employed to elucidate the respective oxidative stress triggers and concomitant amelioration remedies [1,2,3]. A readily available source of antioxidants concerns plant-based natural food products containing secondary metabolites with high radical scavenging potency (non-enzymatic antioxidant groups), and such natural products have received considerable attention in recent years [4,5,6,7]. On a global scale and in the context of global food security, healthy food consumption has been proposed as a global megatrend along with consumer and industry shifts towards sustainably produced food in the frame of a less predictable planet in environmental terms [8,9]. On local scales, several regional (national) native phytogenetic resources have shown considerable potential as sources of promising healthy foods, fitting into global consumer trends [10,11,12,13]. However, despite their recognition in modern times, a large amount of local plant germplasm resources with high antioxidant potential remains neglected and underutilized [12,14], as opposed to extensively utilized plant-based staple food germplasm resources (e.g., legumes and cereals) that are intensively conserved worldwide [15,16].

In general, plants that are used traditionally in ethnobotany but are neglected and underutilized in commercial terms (NUPs) have been shown to convey significant potential in terms of sustainable agronomic exploitation within the Pentagon framework of biodiversity loss, sustainability, food security, climate change, and economic recession [14,17,18,19]. In addition, a wide variety of NUPs can also convey significant phytochemical potential when supported by research results related to health properties that fit into modern trends in pharmacology and phytochemistry. However, modern utilization efforts of such resources (e.g., NUPs and products with high antioxidant potency) usually take place either on a regional basis or on a very small commercial scale [11,14,20]. Nevertheless, NUPs of local food plant germplasm pose a noteworthy source of new materials for the introduction or rediscovery of crops with high nutraceutical potential able to provide excellent and novel sources of healthy food. The latter trend additionally aligns with current consumer trends seeking novel healthy foods produced sustainably and may also be coupled with new options for the income diversification of local producers who currently struggle with the highly increased production cost of mainstream industrial/arable crops; the latter has been further intensified lately by global issues such as the pandemic, or regional ones such as the war in Ukraine [21,22,23]. Novel food crops also including fruits from small wild-growing forest trees and shrubs that are traditionally used at local scales but are currently neglected and underutilized in commercial terms are characterized as neglected and underutilized plant species (NUPs). NUPs offer to date a growing market potential as diversified sources of high-potency antioxidants [12,24,25,26]. Attempts at the local domestication of wild-growing germplasm of small forest fruit tree and shrub NUPs with antioxidant potential for sustainable utilization have been carried out over the last few decades in several regions of the world [27,28,29,30], including Greece [12,24,25]. Additional attempts have been performed on other local endemic NUPs with medicinal-cosmetic, agro-alimentary, and ornamental potential [11,14,31], outlining cases of local endemic NUPs introduced to date as promising new industrial crops [32]. For the successful development of a framework allowing for the establishment of new crops for novel products, targeted applied research efforts are needed, and these should be systematized into distinct and interlinked stages that can provide a multifaceted and comparative evaluation of the overall utilization potential for selected NUPs [24,25].

Species-wise, the current study is focused on four different NUPs: First, *Amelanchier ovalis* (snowy mespilus or serviceberry, Rosaceae) with small (5–15 mm across) crimson-colored pomes that turn black at full maturity; these edible fruits (but also other plant organs) have been reported to contain high levels of anthocyanins and flavanols delivering high antioxidant potency [26,33,34]. In ethnobotanical terms, traditional uses of *A. ovalis* in Greece are rather limited and only scarce fruit consumption directly from the wild has been mentioned by village inhabitants around some of the species’ occurrence areas in Greece. Second, *Cornus mas* (Cornelian cherry, Cornaceae) with deep red, cherry-like fruits (drupes) shows high antioxidant capacity due to high levels of phenolic compounds, vitamin C, and flavonoids, among others [35,36] as well as high free radical scavenging properties [25,37,38,39]. Traditional uses of cornelian cherries in Greece include incorporation in homemade jams or pastries, but the most common is the traditional cornelian cherry liquor [40]. Then, *Rosa canina* (dogrose, Rosaceae) with red rose hips containing antioxidant compounds such as flavonoids, tannins, anthocyanins, and high levels of vitamin C [41], which are associated with the widespread nutritional and pharmaceutical value in terms of antioxidant activity [12,28,42,43,44]. Dogrose has modern culinary uses in Greece, such as syrup for tarts and jams, reflecting its traditional use as an infusion beverage both in Greece and elsewhere [40]. Last, *Sambucus nigra* (elderberry, Viburnaceae) with small, berry-like dark purple fruits shows significant antiviral and antimicrobial potency [29,45] and strong antioxidant properties [46,47]. Elderberries have been traditionally used in some mountain villages of Greece as elaborated food additives for flavor in ice creams, jams, and jellies.

Although *C. mas*, *R. canina*, and *S. nigra* have been traditionally and ethnobotanically used in Greece, the use of *A. ovalis* is quite limited. However, considering the documented phytochemical potential of all the above-mentioned plants, they are not utilized to date as a germplasm resource in terms of sustainable agronomic exploitation, and thus they are considered neglected and underutilized. Therefore, a consolidated framework for the sustainable agronomic exploitation of the Greek native germplasm of the above-mentioned focal NUPs has been brought forward lately, furnishing multifaceted data on their documentation and molecular authentication of different wild-sourced genotypes, along with their phytochemical evaluation, asexual propagation, and ex situ cultivation [12,24,25,26,48,49].

In this context, the scope of this work was mainly two-fold: (a) To build on own relevant studies published previously [12,24,25,26] by adding new information on the evaluation of the ex situ cultivation regarding three of the four focal NUPs, thus compiling respective full datasets for their comparative evaluation; and (b) To determine comparatively the feasibility and readiness timescale for the sustainable exploitation of the focal Greek native NUPs based on established protocols [14] after a meta-analysis of overviewed respective data in four evaluation axes, namely documentation and molecular authentication, phytochemical evaluation, asexual propagation, and ex situ cultivation. The overarching aim was to be able to set forward the consolidated basis for the sustainable agronomic exploitation of the selected Greek native germplasm of NUPs as promising natural sources of antioxidants.

## 2. Results and Discussion

### 2.1. Bridging Extant Research Gaps with Ex Situ Cultivation Trials

The ex situ cultivation trial of Greek native germplasm of *R. canina*, *C. mas*, and *S. nigra* was established in 2020 as a pilot cultivation attempt of wild germplasm; however, no species-specific evaluation report has been published in our own previous studies [12,24,25,26]. Therefore, young trees’ establishment and juvenile growth were recorded for two consecutive growing seasons (2020 and 2021) under different fertilization regimes, including conventional fertilization and a diversified organic fertilization regime, which were applied for the first time on a cultivation trial of wild germplasm.

**Figure 1:**
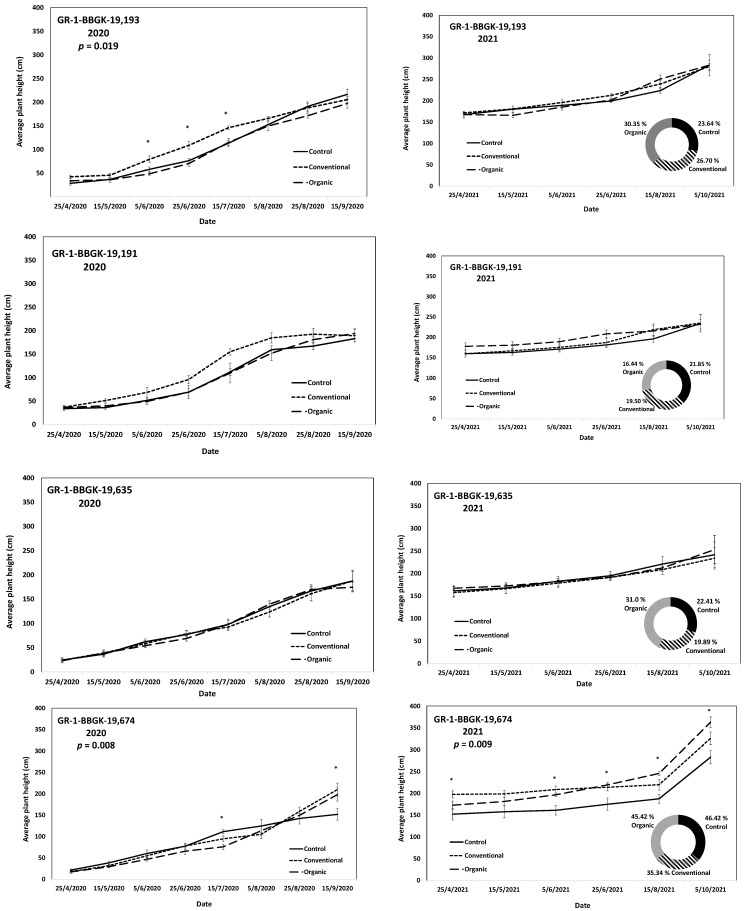
Plant growth patterns expressed as average plant height (cm) for *Rosa canina* genotypes GR-1-BBGK-19,193, GR-1-BBGK-19,191, GR-1-BBGK-19,635, and GR-1-BBGK-19,674 during the first two years of the pilot field trial (2020 and 2021) for the three fertilization treatments applied (control, conventional, and organic). Standard errors of the means are shown on the graphs (*p* < 0.05). The treatment effects over time (within-subjects effects) were assessed via a Repeated Measures ANOVA separately for each genotype and year and were found significant in all cases (*p* < 0.05). In cases where significant fertilization treatment effects (not over time, between-subjects effects) were observed, the respective *p* values are given, coupled with asterisks that denote dates when significant differences between treatments were observed after discreet analyses for each measurement date via Tukey’s HSD mean comparison (*p* < 0.05). In the 2021 graphs per genotype, the rate of juvenile growth expressed as percentage (%) of tree height increase between 6 and 18 months after planting for each fertilization treatment measured independently, is comparatively given in respective circular graphs.

**Figure 2:**
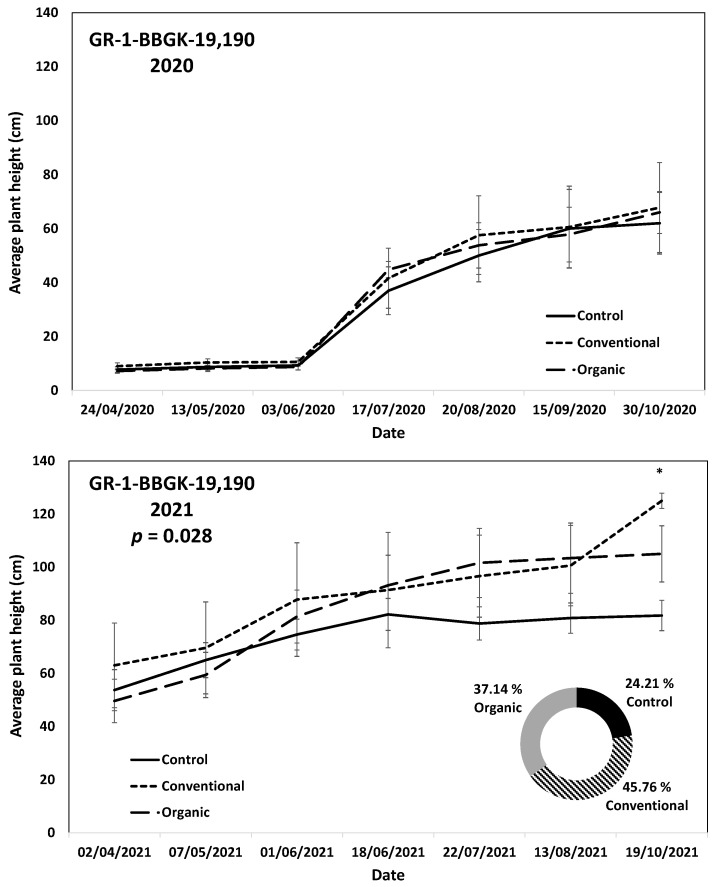
Plant growth patterns expressed as average plant height (cm) for *Cornus mas* genotype GR-1-BBGK-19,190 during the first two years of the pilot field trial (2020 and 2021) for the three fertilization treatments applied (control, conventional, and organic). Standard errors of the means are shown on the graphs (*p* < 0.05). The treatment effects over time (within-subjects effects) were assessed via a Repeated Measures ANOVA separately for each year and were found significant in both cases (*p* < 0.05). In 2021, where significant fertilization treatment effects (not over time, between-subjects effects) were observed, the respective *p*-value is given, coupled with asterisks that denote dates when significant differences between treatments were observed after discreet analyses for each measurement date via Tukey’s HSD mean comparison (*p* < 0.05). In the 2021 graph, the rate of juvenile growth expressed as percentage (%) of tree height increase between 6 and 18 months after planting for each fertilization treatment measured independently, is comparatively given in a circular graph.

**Figure 3:**
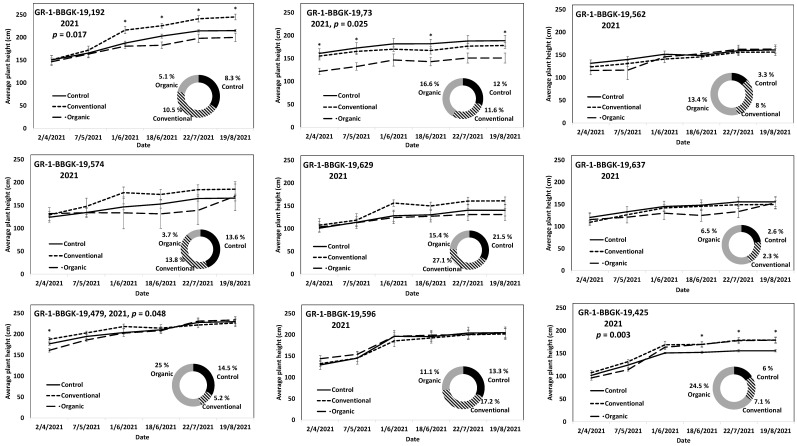
Plant growth patterns expressed as average plant height (cm) for *Sambucus nigra* genotypes GR-1-BBGK-19,192, GR-1-BBGK-19,73, GR-1-BBGK-19,562, GR-1-BBGK-19,574, GR-1-BBGK-19,629, GR-1-BBGK-19,637, GR-1-BBGK-19,479, GR-1-BBGK-19,596, and GR-1-BBGK-19,425 during the second year of the pilot field trial (2021) for the three fertilization treatments applied (control, conventional, and organic). Standard errors of the means are shown on the graphs (*p* < 0.05). The treatment effects over time (within-subjects effects) that were assessed via a Repeated Measures ANOVA separately for each genotype were found significant in all cases (*p* < 0.05). In cases where significant fertilization treatment effects (not over time, between-subjects effects) were observed, the respective *p* values are given, coupled with asterisks that denote dates when significant differences between treatments were observed after discreet analyses for each measurement date via Tukey’s HSD mean comparison (*p* < 0.05). The rate of juvenile growth expressed as percentage (%) of tree height increase between 6 and 18 months after planting for each fertilization treatment measured independently, is comparatively given in respective circular graphs for each genotype.

The results of the ex situ cultivation trial for Greek *R. canina* have shown differences in juvenile tree growth among the tested genotypes throughout the applied treatments during the first 18 months after planting, corresponding to two growing seasons, 2020 and 2021 (Figure 1). Over the above period, the genotypes GR-1-BBGK-19,191 and GR-1-BBGK-19,635 showed no differences among fertilization treatments, reaching by the end of the 2021 season, 2.32–2.35 m and 2.34–2.53 m respective plant heights among treatments (Figure 1). At the same time, the increase of the rate of height between the 2020 and 2021 growing seasons (6–18 months after planting) reached 19.5 and 21.8% in the conventionally fertilized and control trees, respectively, of the genotype GR-1-BBGK-19,191; whereas in the GR-1-BBGK-19,635 genotype, the organic fertilization showed the highest (31% in average) increase rate between 6 and 18 months after planting (Figure 1). The genotype GR-1-BBGK-19,193 showed higher growth rates under the conventional fertilization regime during June and July 2020 but with no further difference until the end of the 2021 season (*p* < 0.05, Figure 1), which resulted in a higher rate of height increase by the organic fertilization (30.3%) between 6 and 18 months after planting (Figure 1). The genotype GR-1-BBGK-19,674 demonstrated the highest growth patterns among all tested genotypes with significantly higher trees under conventional and organic fertilization compared to the control, reaching at the end of the 2021 season 3.3 m and 3.6 m height, respectively (*p* < 0.05, Figure 1), with a 45.4% height increase rate of organic fertilization. In mature trees of the cultivated germplasm of almonds (Rosaceae), organic fertilization via green manure in combination with minimum tillage can potentially enhance growth and production under semi-arid conditions [50]. Similarly, organic fertilization in olive trees (*Olea europaea* L.) has been suggested to enhance soil fertility positively, thus affecting production [51]. On the other hand, conventional fertilization on the domesticated germplasm of pears and almonds should be applied up to specific thresholds to avoid excessive N concentrations in the soil [52,53]. Nonetheless, the current results suggested that organic fertilization in *R. canina* seemed to enhance juvenile growth. On the contrary, the Greek *C. mas* genotype GR-1-BBGK-19,190 (which was assessed during the two growing seasons (2020 and 2021) herein) showed a 45.7% height increase rate between 6 and 18 months after planting under the conventional fertilization treatment, which resulted in significantly greater average tree height at the end of 2021, with 1.25 m compared to 0.82 m and 1.05 m in control and organic fertilization, respectively (*p* < 0.05, Figure 2). In the case of *S. nigra*, a variation in fertilization effects on growth among genotypes was observed (Figure 3), corroborating with variation among the same genotypes on asexual propagation potential, as previously observed [24]. In five out of the nine tested genotypes, organic fertilization showed higher juvenile growth rates, with the remaining four genotypes showing higher growth rates under conventional fertilization (Figure 3). The *S. nigra* genotype GR-1-BBGK-19,192 showed the highest tree growth under conventional fertilization, with 2.45 m average tree height at the end of 2021, and this genotype also showed the best performance in asexual propagation potential (Figure 3), [24]. The second-best field trial performance in terms of tree height was observed in the genotype GR-1-BBGK-19,479 which had the highest juvenile growth rate under organic fertilization (Figure 3). Thus, the current data suggested that conventional fertilization performed comparatively better in *C. mas* than in *R. canina*, but under much lower final tree heights, thus outlining species-specific differences.

The results on *S. nigra* during the 2021 trial outlined variation among genotypes (Figure 3). The *S. nigra* genotypes during the first year of the field trial (2020) also showed similar genotype variation, with conventional fertilization showing higher tree establishment in general, but with similar tree juvenile growth rates among conventional and organic fertilization [24]. Additional, extant data on the cultivation trial of the Greek wild germplasm of *A. ovalis* showed higher tree establishment potential in terms of tree height at 18 months after planting under conventional fertilization, but with a higher tree growth rate between 6 and 18 months after planting under organic fertilization [26]. Furthermore, differences among the focal species were also observed in the onset of fruit production, which in *R. canina* and in *S. nigra* took place in the second year after planting (2021), whereas in *C. mas* no fruit production was evident for the duration of the current study. Therefore, further evaluation is warranted by the current results along with trees’ aging as they enter their reproductive maturity, thus considering additional factors such as the physiological balance of trees [54] as affected by fertilization type [55].

### 2.2. From Wild Species to Successfully Domesticated and Sustainably Managed New Crops

**Figure 4:**
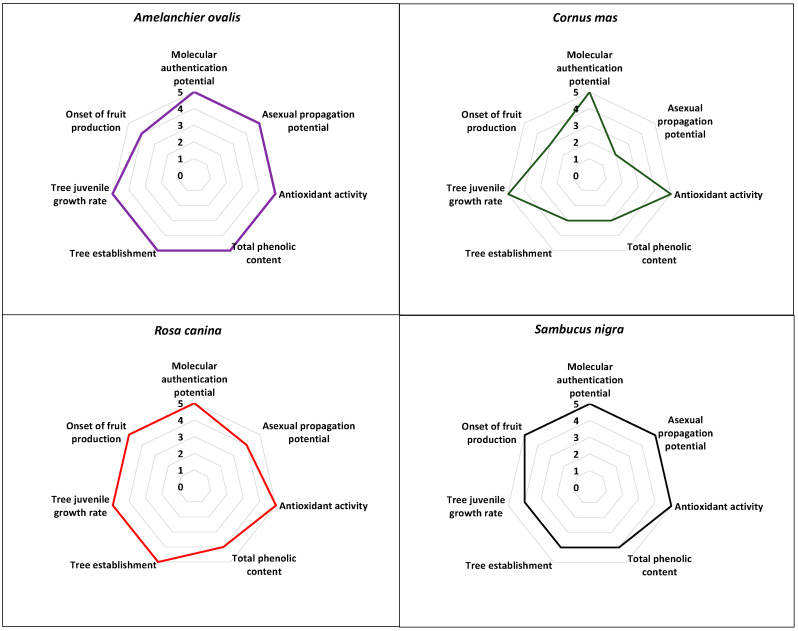
Comparative evaluation of the multifaceted potential of the four Greek native, neglected, and underutilized species (*Amelanchier ovalis*, *Cornus mas*, *Rosa canina*, and *Sambucus nigra*).

The comparative evaluation results regarding the multifaceted potential of the herein studied NUPs outlined the very high scores associated with the cases of the Greek *A. ovalis*, *R. canina*, and *S. nigra* with 34–97.1%, 33–94.3%, and 32–91.4%, respectively (Figure 4; Supplementary Material Table S1) followed by *C. mas* with a score of 26–74.3% (Figure 4; Supplementary Material Table S1). Therefore, all four NUPs evaluated herein have shown excellent multifaceted potential for agronomic exploitation (Figure 4).

Firstly, the establishment of a unique genetic identity was successful for the population samples (genotypes) of all focal NUPs studied herein (e.g., [12,24], Figure 4), which, in theory, can be agronomically exploited in terms of branding and marketing, contributing to products’ uniqueness, i.e., protected geographical indication (PGI products). Generally, it is known that DNA barcoding, apart from complementing classical morphological identification of population samples, can also elucidate the phylogenetic relationships of geographically separated genotypes, thus providing information on the genetic identity of neglected or underutilized germplasm [12,24,56].

Secondly, all four NUPs evaluated herein have shown excellent potential in terms of fruit antioxidant activity (%RSA), albeit with a variation in total phenolic content (TPC, mgGAE/100 g) (Figure 4; Supplementary Material Table S1). Undoubtedly, the antioxidant potential is of pivotal importance for the sustainable exploitation of these NUPs. The comparative evaluation of the antioxidant potential as evaluated herein may outline the very high antioxidant potency among Greek population samples of all focal NUPs, thus indicating the strong nutraceutical value of the studied germplasm. It should be noted that the high antioxidant potential exhibited by the focal NUPs studied herein has been evaluated under Mediterranean climatic conditions with some continental features characterizing the area of Thessaloniki in northern Greece [25,26]. This, however, would warrant further research regarding the response of antioxidant potential under different environments. In any case, similar results have also been reported in other studies evaluating the same NUPs from different regions [27,28,29,30,57], thus indicating that the high antioxidant potency may be species-specific. In general, plant-based secondary metabolites with known high free radical scavenging activity due to phenolic compounds, flavonoids, or high vitamin C content can ameliorate the effects of human oxidative stress [58].

Propagation-wise, the current results showed very high asexual propagation potential via the rooting of cuttings for *A. ovalis* and *S. nigra* followed by *R. canina*, with propagation performance above commercially accepted standards [59], (Figure 4; Supplementary Material Table S1). For Greek *C. mas*, however, the propagation potential was comparatively lower due to the high level of variation among genotypes from <5% to >65% (Figure 4; Supplementary Material Table S1), [25]. Similar levels of variation in the rooting capacity of cuttings among wild genotypes have also been documented in other fruit tree species of the genus *Prunus* [60]. The asexual propagation is known to facilitate the bulk production of initial plant material for ex situ conservation and further experimentation securing, at the same time, the retention of desirable agronomical traits among the genotypes of focal species, such as antioxidant potential. In general, the availability of high-volume, high-quality propagation material is essential for any sustainable agronomic exploitation attempt [12,24,25,26]. As such, the development of effective asexual propagation protocols has been the focus of relevant studies conducted on the species studied herein [61,62].

In terms of ex situ cultivation of the wild germplasm, three of the four species showed excellent potential in tree juvenile growth rate, apart from *S. nigra*, which was found scoring one class lower (Figure 4), while for tree establishment, *A. ovalis* and *R. canina* showed excellent potential, followed by *S. nigra* and *C. mas*. Regarding the onset of fruit production, *R. canina* and *S. nigra* produced fruits as early as the 2nd season after planting, *A. ovalis* produced fruits in the 3rd season, whereas *C. mas* did not produce fruits by the 3rd season (Figure 4). Although the focal species studied herein, except for *A. ovalis*, have documented cultivations elsewhere [57,63,64,65], the extant data presented and sourced herein provide information for the first time on trial field cultivation of the Greek native germplasm of all four focal species, albeit at a pivotal level [24,26]. Nonetheless, the comparative evaluation results obtained may showcase the high field cultivation potential of all focal NUPs in terms of tree establishment in Greece with an effective tree juvenile growth rate, with only *C. mas* showing slightly lower scores of tree establishment and the onset of fruit production.

Following the ever-accumulating scientific knowledge on climate change and global food security under increasing global population trends, the contemporary applied research lines tend to shift from traditional demand for increased food production at all costs towards the need for increased production of healthy food that is being produced in a sustainable and friendly manner in environmental terms [9,66,67]. From this perspective, the research focusing on NUP germplasms to be rediscovered or introduced as new alternative crops are considered of high importance. In this novel research line, the selection of plant species with high natural resistance and nutraceutical potential requiring minimum amounts of inputs that are traditionally used locally but are commercially and agronomically neglected and underutilized offers to date new possibilities for the introduction of alternative crops (upon domestication), utilizing sustainably local or regional germplasm pools in the frame of current global research trends and priorities [10,68,69,70].

### 2.3. Stepping Forward for the Sustainable Exploitation of the Studied NUPs

Another innovative evaluation methodology has been applied herein to explore the feasibility and readiness timescale for the sustainable exploitation of the focal species and their value chain creation [14]. Considering the obtained results, *R. canina* showed the highest feasibility (72%, Table 1) and an already achieved readiness timescale for its sustainable exploitation (Table 2). Similarly, *S. nigra* also showed a feasibility score for sustainable exploitation of 70.8% and an already achieved readiness timescale (Table 1 and Table 2). These two cases may highlight that all necessary prerequisites and conditions for value chain creation can be easily achieved because all research gaps have been effectively bridged [11,14,31]. Furthermore, *C. mas* and *A. ovalis* scored 62.5% and 55.5% in terms of feasibility for sustainable exploitation, respectively, thus, both can be classified as having an achievable readiness in the short-term (Table 1 and Table 2). Combining these assessments with the results of the comparative evaluation regarding the multifaceted potential of the Greek native germplasm as discussed above, it can be mentioned that significant new opportunities emerge for these highly potent nutraceutical NUPs as new crops.

The herein studied NUPs have ethnobotanical uses that can be integrated with the latest research on their phytochemical potential, thus fitting modern trends in pharmacology and phytochemistry. In addition, considering the results of the work herein, several important issues have been addressed for the studied NUPs, such as the documentation of their high antioxidant potential, the extant availability of suitable propagation material of consolidated identity, the existing cultivation protocols described in brief herein, the available (or potentially available) commercial products and associated market needs, the fact that no legal restrictions are associated with lawful access to these materials, and the benefit sharing mechanisms in place (see EU Directive 511/2014). Considering these issues altogether, it can be suggested that upscaling to sizable commercial cultivation settings is currently feasible for these NUPs in a relatively short time frame, which indeed could apply to the Greek market as well. However, from a strict horticultural point of view, further applied research may be needed in terms of cost calculation for orchard establishment, calculation of the cost of production, possible pests and disease resistance in man-made settings, and definition of the length of the productive life of each species. Such aspects have been previously outlined in relevant studies attempting to upscale new fruit tree crops [78,79,80] and can certainly further consolidate the estimated herein feasibility and readiness of the studied germplasm for effective sustainable exploitation and effective value chain creation. Further issues such as the deleterious effects of toxic secondary metabolites of *S. nigra* fruits should also be taken into consideration during the cultivation/production/processing processes [81], while chemical analysis and certification legal instruments should also be put in place. Extant EU and Greek certification mechanisms do exist for assessing pesticide residues in fruit crops such as table grapes, which could also be exploited for other fruit crops such as those studied herein. In addition, at the local scale of Greece, certain issues and conditions related to the estimated readiness timescale for sustainable exploitation should be further addressed. These include concomitant difficulties in value chain creation for novel crops derived from NUPs and employ the exploration of the possibility to exploit extant distribution channels for them in terms of similarity of fruit typology, fruit size, texture, and post-harvest self-life. Such issues cannot easily be resolved in Greece for native NUPs such as the ones studied herein because these novel crops are still in their infancy and to a significant extent all related agro-processing issues are ill-explored, thus discouraging potential growers from upscaling from pilot to sizable levels. Therefore, bold decisions seem to be necessary for anyone involved in such attempts, and stakeholder attention is required to further address such issues [11,14,31]. Indicatively, dogrose hips and/or cornelian cherries and their products, such as infusions, jams, or liquors, are currently traded only from a few producers in Greece and elsewhere, involving usually local family businesses performed at small scale, while the designated origin of the raw materials used largely remains unclear, either imported or unsustainably sourced from the wild. Nevertheless, existing value chains and value chain creation or enhancement attempts should employ the exploitation of the extant distribution channels of other mainstream stone fruits or pomes that could be easily used for the types of small fruits studied herein. Marketing campaigns and smart branding based on the fundamental advantage of the current fruits of the studied NUPs in terms of highly documented antioxidant potential should precede any upscaling effort.

## 3. Materials and Methods

### 3.1. Comparative Evaluation of the Multifaceted Potential of the Greek Native Germplasm

#### 3.1.1. Authorized Access to the Wild-Growing Greek Native Germplasm and Documentation

Under the auspices of the Eco-Variety research project (2018–2022), a total of 54 geographically separated population samples of variable-type material (cuttings and stem parts as propagation material, leaves for DNA analysis, ripe fruit samples) from the four small tree/shrub focal NUPs were taxonomically identified in situ [82]. The specimens were collected using a special collection permit of the Institute of Plant Breeding and Genetic Resources (IPBGR), Hellenic Agricultural Organization Demeter (ELGO-Dimitra) (Permit 82336/879 of 18 May 2019 and 26895/1527 of 21 April 2021) issued by the Greek Ministry of Environment and Energy. The collections were made during targeted botanical expeditions across four regions of northern Greece. Each collected and taxonomically identified population sample (genotype) was allocated a unique IPEN (International Plant Exchange Network) accession number given by the Balkan Botanic Garden of Kroussia, IPBGR, ELGO-Demeter. Following each expedition, the sampled material was sorted, documented, and handled, as described in Karapatzak et al. [25].

#### 3.1.2. Different Axes for the Evaluation of the Multifaceted Potential of the Focal NUPs

The data evaluated herein were produced through multi-year systematized research (still ongoing) for each of the focal NUPs, namely *A. ovalis* [26], *C. mas* [25], *R. canina* [12], and *S. nigra* [24,81]. The produced results in the above-mentioned studies have been used herein to assess in a comparative way the multifaceted potential of each taxon in distinct and interlinked evaluation axes as follows:

1. Molecular authentication potential: The establishment of a distinct genetic identity as an essential part of the documentation of the collected population samples was conducted via DNA barcoding using the ITS2 barcode region and respective phylogenetic relationships [12,24,25,48]. The work resulted in the genetic fingerprint of all documented Greek genotypes compared to other known genotypes of the same species deposited at the NCBI originating from other regions [12,24,25,48].
2. Phytochemical potential: The phytochemical potential of the documented Greek genotypes of each focal species was assessed on the basis of available ripe fruit samples via four basic indicators of antioxidant potency, namely total phenolic content (TPC), antioxidant activity (AA), total flavonoids (TF), and vitamin C content for *R. canina* [12], *C. mas* [25], *A. ovalis* [26], and *S. nigra* [49]. The extant data sourced herein consider antioxidant activity (AA) and total phenolic content (TPC) measured among different population samples (genotypes) of all focal NUPs (Table 3).
3. Asexual propagation potential: The development of effective asexual propagation protocols was considered necessary for securing the production of initial material, safeguarding the steady transfer of the agronomical traits of each evaluated genotype. Asexual propagation via cuttings was attempted in population samples directly collected from the wild to facilitate ex situ conservation on one hand, and to secure the establishment of mother plant stock material for further experimentation, on the other hand, for all focal species [12,24,25,26]. The extant data sourced herein considers the rooting frequencies delivered by the experimentally developed protocols among different population samples (genotypes) of all focal NUPs (Table 3).
4. Cultivation potential: Based on the results of the above attributes, selected genotypes from each focal species were established at an orchard-type pilot cultivation trial in 2020 [24,26], where the development of trees in terms of plant height coupled with fruit production under different fertilization regimes was assessed, aimed at the development of customized cultivation protocols for each species (Table 3). Tree growth was measured continually for three years (2020–2022) and is still ongoing for *A. ovalis* [26], *S. nigra* [24], *R. canina*, and *C. mas*. The results of the ex situ trial cultivation of *R. canina* and *C. mas* are presented for the first time herein, as well as the 2nd year’s data for *S. nigra*. The ex situ cultivation data for *R. canina*, *C. mas*, and *S. nigra* were obtained from a field cultivation trial that took place on the grounds of the IPBGR, ELGO-Dimitra in Thermi, metropolitan Thessaloniki, Greece (40.534934 N, 23.002401 E, 40 m elevation). The trial commenced in March 2020 when cutting-originated plants were planted in a medium composition loamy soil with 34% clay, 48% sand, and 1.37% organic matter. The climatic conditions at the trial site were generally Mediterranean with continental features. Four genotypes of *R. canina,* one genotype of *C. mas*, and nine genotypes of *S. nigra* were tested against three fertilization regimes: no fertilization (control), conventional crop fertilization rich in N, and organic crop fertilization consisting of standardized organic fertilizers containing, among others, a plethora of organic acids, amino acids, humic acid, and nitrogen, and applied gradually over each growing season. The fertilization regimes were empirically designed and were applied throughout the first two growing seasons until 18 months after planting in 2020 and 2021 (as described in Karapatzak et al. [24,26]). The juvenile growth of young trees was assessed via measurement of plant height (cm) at regular intervals during the first 18 months after planting, allowing the estimation of a growth rate (%) for a period from 6 to 18 months. In addition, the onset of fruit production was recorded. Therefore, the data on field cultivation potential herein include the following for each NUP: (a) tree establishment in terms of juvenile tree height reached at 18 months after planting coupled with juvenile tree development in terms of increase (%) in height from 6–18 months after planting, and (b) the onset of fruit production in terms of temporal distance from initial planting (i.e., after how many years or growth seasons after planting trees started to fruit).

Three distinct datasets are presented herein for the first time, i.e., the ex situ trial cultivation tree growth data for *R. canina*, *C. mas*, and *S. nigra*. For the ex situ trial cultivation, a completely randomized design was followed, which, for the case of *R. canina*, included four genotypes, whereas for *S. nigra* nine genotypes were tested under three distinct fertilization treatments (including control), with five replicate plants per treatment. Similarly, for the case of *C. mas*, the trial employed one genotype for 0–18 months after planting under the same experimental design with *R. canina* and *S. nigra*. The treatment effects on plant height data measured over time on the same experimental units were assessed through repeated measures ANOVA for each genotype, during each growing season separately (a = 0.05) to assess whether the observed differences resulted over time or were purely due to the fertilization treatment effects or both. In addition, a GLM-ANOVA for fertilization treatments was conducted discreetly for each genotype across each measurement date coupled with mean comparison through Tukey’s HSD post hoc test (a = 0.05) to pinpoint any specific treatment differences at different genotypes and different dates. All analyses were conducted using the IBM-SPSS 23.0 software (IBM Corp., Armonk, NY, USA), and graphs were drawn using Microsoft Excel.

#### 3.1.3. Scoring of Multifaceted Potential with Attributes after Comparative Meta-Analysis

All four focal Greek native NUPs were comparatively evaluated herein based on the above-mentioned axes and source data (Table 3). The extant data considered several population samples (genotypes) from each NUP, the results of which were summarized and expressed as average values among the examined genotypes for each species. The scores assigned to data were summarized for each species independently, and the escalation of scoring was determined considering the extant quantitative data available (Table 4). For three of the four evaluation axes (phytochemical potential, asexual propagation potential, and cultivation potential), a five-fold scoring scale was determined a priori based on purely quantitative standards consisting of consecutive numerical ranges from lowest to highest (Table 4). The directionality of scoring favored the most effective results (highest measurement results associated with the highest scores). For the molecular authentication potential, the scoring scale was only two-fold (a distinct genetic identity either established or not) for each population sample of each focal species (Table 4).

### 3.2. Feasibility and Readiness Timescale Evaluation for Sustainable Exploitation

For the evaluation of the feasibility of sustainable agronomic exploitation, we used the methodological scheme developed by Krigas et al. [14]. As a prerequisite, 12 designated attributes were assessed per taxon using quantitative and/or qualitative scoring scales per attribute to assess extant data with a directionality of scoring favoring the most desirable traits [14]. The evaluation attributes included the following: the existence of current cultivations (two scores, either 0 or 6); threat category status (scoring 0–6); species protection status (four scores: 0, 4, 5, or 6); ex situ conservation status (scoring 0–6); distribution range (scoring 0–6); existing commercial products (scoring 0–6); known propagation protocols (four scores: 0, 3, 5, or 6); vegetative propagation success achieved (scoring 0–6); seed germination success achieved (scoring 0–6); known cultivation needs (four scores: 0, 3, 5, or 6); extant cultivation protocols (scoring 0–6); and known water demands (four scores: 0, 1, 3, or 6). Several of these designated attributes have been used to assess targeted prerequisites that should be met to facilitate the sustainable exploitation potential of a NUP in economic sectors such as known propagation protocols and known cultivation protocols as well as existing successfully marketed products [14]. On the other hand, further designated attributes assessed targeted species-specific aspects that can potentially enhance possible branding and marketing issues, thus outlining the uniqueness of products designed, such as species endemism related to exclusive branding potential and distribution range related to opportunities for protected designation of origin (PDO) products [14]. The respective overall percentage (%) was calculated for each evaluated species stemming from the individual scoring results per attribute against summed maximum values for all attributes, thus showing the highest theoretical scoring that could be achieved (maximum possible score); in this way, the overall score achieved may illustrate each species’ feasibility potential for sustainable agronomic exploitation compared to the ideal (optimum) one [14]. Based on the results of the feasibility evaluation for sustainable agronomic exploitation, each of the four focal NUPs was assigned to a designated category in terms of its readiness timescale for sustainable agronomic exploitation according to the necessary conditions met, as defined by Krigas et al. [14].

## 4. Conclusions

This study presented for the first time a comparative and multifaceted evaluation approach for the sustainable agronomic potential of Greek native germplasm of small tree/shrub species as natural sources of antioxidants. The studied focal species are traditionally used in Greek ethnobotany but are currently underutilized in agronomic and commercial terms. Overall, based on the results of all evaluations for sustainable exploitation conducted herein, the Greek native germplasm of *R. canina* shows excellent perspectives in terms of multifaceted potential, feasibility, and readiness timescale for sustainable exploitation, and the same holds true for *S. nigra* with an equally high multifaceted potential or feasibility and readiness timescale for sustainable exploitation. Furthermore, the Greek native germplasm of *C. mas* and *A. ovalis* seems to share similarly high multifaceted potential but a comparatively inferior feasibility and readiness timescale for sustainable exploitation, which, however, can reach achievable perspectives in the short term. The approach applied herein enables the critical evaluation of focal NUPs under a wide set of attributes, thus illustrating a multi-dimensional picture of current perspectives and concomitant challenges that can facilitate in turn the decision-making procedures, the prioritization strategies, the shaping of further applied research attempts, and the channeling of commercial utilization efforts. In addition, from a long-term perspective, targeted breeding efforts based on the extant datasets documented herein could further enhance the course from wild-growing plant species that are neglected and underutilized to successfully domesticated and sustainably managed selections of suitable genetic materials for new crops.

## Figures and Tables

**Table 1:** Overview of the individual scores against 12 attributes designated by Krigas et al. [14], the overall score achieved per species out of a maximum total score of 72, and respective feasibility percentage (%) along with the literature sources used in the evaluation of feasibility for sustainable exploitation regarding the four focal species studied herein (*Amelanchier ovalis*, *Cornus mas*, *Rosa canina*, and *Sambucus nigra*).

Evaluation Attribute	*Rosa canina*	*Sambucus nigra*	*Cornus mas*	*Amelanchier ovalis*	Data Sources
Existing cultivations	6	6	6	6	[24,26,57,63,65,71,72] ex situ cultivation data furnished herein
Threat category	1	1	1	0	IUCN https://www.iucnredlist.org/ (accessed on 27 January 2023)
Protection status	0	0	0	0	Greek Presidential Decree 67/1981, EU Directive 92/43/EEC, CITES
Ex situ conservation	6	6	6	6	BGCI.org/Plant Search
Distribution	0	0	0	1	https://portal.cybertaxonomy.org/flora-greece/intro (accessed on 27 January 2023)
Commercial products	6	6	5	1	[38,63,73,74]
Known propagation	6	6	6	6	[12,24,25,26,63,75,76,77]
Vegetative propagation Success	6	6	6	6	[12,24,25,26]
Seed germination success	6	5	0	0	[63,75,76,77]
Cultivation needs	6	6	6	6	[24,26,57,63,64,71] ex situ cultivation data furnished herein
Existing cultivation protocols	6	6	6	5	[24,26,65,71,72] ex situ cultivation data furnished herein
Water demand	3	3	3	3	[24,26,64]
Total score (respective %)	52 (72.2)	51 (70.8)	45 (62.5)	40 (55.5)	

**Table 2:** Evaluation of the readiness timescale for sustainable exploitation of the four focal species studied herein (*Amelanchier ovalis*, *Cornus mas*, *Rosa canina*, and *Sambucus nigra*) according to the conditions and criteria designated by Krigas et al. [14].

Focal Species	*Rosa canina*	*Sambucus nigra*	*Cornus mas*	*Amelanchier ovalis*
Readiness timescale	Already achieved (>70%)	Already achieved (>70%)	Achievable in short-term (>55–70%)	Achievable in short-term (>55–70%)
Up-scaling to address commercial demand	Upon request	Upon request	Easy	Easy
Availability of propagation material	Readily available	Readily available	Potentially easy	Potentially easy
Possibility to overcome legal restrictions (in relation to interest)	No obstacle (strong)	No obstacle (strong)	Feasible (increased)	Feasible (increased)
Overview of extant research gaps	No research gaps	No research gaps	Minor research gaps	Minor research gaps
Estimated attraction of new producers-retailers	Extant	Extant	Feasible	Feasible
Estimated difficulty for value chain creation	Extant	Extant	Need for enhancement	Need for enhancement
Estimated exploitation of distribution channels	Extant	Extant	Feasible	Feasible

**Table 3:** Overview of extant documentation regarding the multifaceted potential (documentation and molecular authentication, phytochemical potential of fruits, asexual propagation potential via rooting of cuttings, and ex situ trial cultivation potential) of the selected Greek native, neglected, and underutilized germplasm (genotypes) and contribution of the current study in dataset completion per focal species.

Focal Species	Documentation and Molecular Authentication Potential (Genotypes)	Phytochemical Potential (Genotypes)	Asexual (Cuttings) Reproduction Potential (Genotypes)	Ex Situ Trial Cultivation Potential/Yearly Data (Genotypes)	Sources
*Amelanchier ovalis*	Yes (10)	Yes (1)	Yes (1)	Yes/3 years (1)	[26,48]
*Cornus mas*	Yes (18)	Yes (14)	Yes (18)	No */(1)	[25]
*Rosa canina*	Yes (12)	Yes (7)	Yes (3)	No */(4)	[12]
*Sambucus nigra*	Yes (14)	Yes (4)	Yes (9)	Yes/1 year (9) **	[24,49]

* Yes with 2 years of data presented in this study; ** 2nd year data presented in this study.

**Table 4:** The methodological scheme used to illustrate comparatively the multifaceted potential of the four Greek native species studied herein with the scoring of seven attributes in four evaluation axes (molecular authentication potential, phytochemical potential, asexual propagation potential, and ex situ cultivation potential) based on available respective data [12,24,25,26,48,49] and data furnished herein (see Table 3).

Score: Designated Potential	Molecular Authentication Potential	Asexual Propagation Potential (% Rooting)	Phytochemical Potential		Field Cultivation Potential		
			Antioxidant Activity (AA, %RSA)	Total Phenolic Content (TPC, mgGAE/100 g)	Tree Establishment: Juvenile Tree Height (m) at 18 Months after Planting	Tree Juvenile Growth Rate: (%) Increase in Height from 6–18 Months after Planting	Onset of Fruit Production
0: No data/not evaluated	Non genetically distinct populations	<5%	<5%	<20	Not established/failed to grow	Not established/failed to grow	No fruit production
1: Very low potential	-	5–20%	5–20%	20–50	<0.5	<5	>5th year
2: Low potential	-	>20–30%	>20–40%	>50–80	0.5–1	5–10	5th year
3: Average or commercially accepted potential	-	>30–50%	>40–65%	>80–100	>1–1.5	>10–15	>3rd year
4: High potential	-	>50–70%	>65–85%	>100–150	>1.5–2	>15–20	3rd year
5: Very high potential	Genetically distinct populations	>70%	>85%	>150–200	>2	>20	As early as the second year
Possible choices	0 or 5	0–5	0–5	0–5	0–5	0–5	0–5

## Data Availability

All data supporting the results of this study are included in the manuscript and/or Supplementary Material, and datasets are available upon request.

## Supplementary Materials

The following supporting information can be downloaded at: https://www.mdpi.com/article/10.3390/plants12081642/s1, Table S1: Scoring results regarding the evaluation of the multifaceted potential of Greek native germplasm (*Amelanchier ovalis*, *Cornus mas*, *Rosa canina*, and *Sambucus nigra*) as natural sources of antioxidants calculated against seven attributes in four evaluation axes designated herein (documentation and molecular authentication, phytochemical evaluation, asexual propagation, and ex situ cultivation). The respective average values (±SD) of the summarized extant data regarding the selected genotypes examined (n) per attribute are given in parentheses next to each score per axis. For the molecular authentication potential, the number of successfully authenticated distinct genotypes against all genotypes collected is given as ratio, whereas for the onset of fruit production the number of genotypes coupled with the season they started to fruit after initial planting is given. For individual scores, see Table 2.
