# Supplementary material for: A Multifaceted Evaluation Approach for Greek Native Neglected and Underutilized Forest Fruit Trees and Shrubs as Natural Sources of Antioxidants: Consolidating the Framework for Their Sustainable Agronomic Exploitation
Source: Plants (Basel). 2023 Apr 13;12(8):1642. doi: 10.3390/plants12081642 (PMC10147030; doi:10.3390/plants12081642)
Supplement: Supplementary file 1 [file plants-12-01642-s001.zip › plants-2254221-supplementary.pdf]

A multifaceted evaluation approach for Greek native neglected and underutilized forest fruit trees and shrubs as natural sources of antioxidants: Consolidating the framework for their sustainable agronomic exploitation

Eleftherios Karapatzak, Olga Dichala, Katerina Papanastasi, Ioannis Manthos, Ioannis Ganopoulos, Antonis Karydas, Anastasia V. Badeka, Ioanna S. Kosma, Dimitris Kyrkas, Paraskevi Yfanti, Nikolaos Nikisianis, Giorgos Patakioutas, Eleni Maloupa and Nikos Krigas

Supplementary Material Table S1: Scoring results regarding the evaluation of the multifaceted potential of Greek native germplasm (*Amelanchier ovalis*, *Cornus mas*, *Rosa canina* and *Sambucus nigra*) as natural sources of antioxidants calculated against seven attributes in four evaluation axes designated herein (documentation and molecular authentication, phytochemical evaluation, asexual propagation, and *ex situ* cultivation). The respective average values (±SD) of the summarized extant data regarding the selected genotypes examined (n) per attribute are given in parentheses next to each score per axis. For the molecular authentication potential, the number of successfully authenticated distinct genotypes against all genotypes collected is given as ratio, whereas for the onset of fruit production the number of genotypes coupled with the season they started to fruit after initial planting is given. For individual scores see Table 4.

Focal species	Molecular authenticat ion potential	Asexual propagation potential (% rooting)	Phytochemical potential		Field cultivation potential			Total score (percentage)	Data sources
			**Antioxidant activity (AA, %RSA)**	**Total phenolic content (TPC, mgGAE/100g)**	**Tree establishment:** juvenile tree height (m) at 18 months after planting	**Tree juvenile growth rate:** (%) increase in height from 6 – 18 months after planting	**Onset of fruit production**		
Amelanchier ovalis	5 (4/10)	5 (83.3, n=1)*	5 (93.13, n=1)	5 (199.65, n=1)	5 (2.04, n=1)	5 (41.6, n=1)	4 (1, 3rd season)	34 (97.1%)	[26,48]
Cornus mas	5 (8/18)	2 (21.1±19, n=18)	5 (85.6±12.41, n=14)	3 (96.8 ±106.7, n=14)	3 (1.25, n=1)	5 (45.8, n=1)	3 (1, >3rd season)	26 (74.3%)	[25]
Rosa canina	5 (9/12)	4 (64.1±13.04, n=3)	5 (94.53±2.59, n=7	4 (101.71±48.65, n=7)	5 (2.83±0.56, n=4)	5 (32.4±10.23, n=4)	5 (4, 2nd season)	33 (94.3%)	[12]
Sambucus nigra	5 (9/14)	5 (97.2±8.33, n=9)	5 (86.3±4.88, n=4)	4 (135.2±70.32, n=4)	4 (1.9±0.31, n=9)	4 (17.2±7.04, n=9)	5 (9, 2nd season)	32 (91.4%)	[24,49]

* No standard deviation is given in cases where only one genotype was selected and examined (n=1)
